# The Hospital Problem and the Prince's Fund

**Published:** 1897-02-20

**Authors:** 


The Hospital

A Journal of

The Medical Sciences and Hospital Administration,

Vol. XXI.—No. 543.] [Feb. 20, 1897.

### THE HOSPITAL PROBLEM AND THE PRINCE'S FUND.

The Prince of Wales' Fund for the hospitals, which is to commemorate for majestic London the sixtieth year of the reign of Queen Victoria, is rapidly attaining to a position of assured success, and will in all probability bring in its train one other result almost as important as the establishment of the financial stability of our great medical charities. It will finally and entirely complete in the mind of the whole thinking public the conviction that the hospital world is truly and indeed a great world, and is worthy of being legislated for and administered by the best legislative and administrative capacity of the times. There are two sets of persons who now control the destinies of hospitals, namely, medical men, and a strictly limited number of subscribers who are also lay managers. When to the influence of these is added that of the permanent officials, there is visible before our eyes the whole of the forces upon which hospitals can now depend for laws and life.

Now this has never been our conception of ideal perfection for the hospital system. We have seen, and we have maintained, that all classes should assume positions of definite responsibility towards medical and charitable relief; they should, in a word, be subscribers, not of money alone, but of personal interest and personal thought. If, or rather, when the Prince's Fund is completed, the question of its administration will inevitably be raised; and it is certain as certain can be that the momentum of such a fund will be such that the whole hospital world will be definitely moved from its traditional moorings, and that the great ship will have to some more or less considerable extent to sail on a new tack. Can this be doubted by any man of experience and thought?

Then, if that be so, there are two public movements now on foot the leaders of which may well be invited to pause. As a matter of fact they must pause, for circumstances, which are in their nature irresistible, are putting an impassable barrier in their way. Those two movements are, of course, the effort now being made to establish on certain narrow lines a Central Hospitals Board, and another effort on still narrower and still less representative lines, to bring about Hospital Reform. We say not a word against either of the ideas which have taken these concrete forms. A board for all London hospitals, rightly constituted, might be of incalculable service to medical charity and education; might, indeed, and probably would, solve the "problem of the hospitals." If that should prove to be so, the other separate movement, the movement for hospital reform, would at once lose its *raison d'être;* for the rightly constituted board would, as one of its first duties, set to work to bring about every reform in every department which the practical necessities of the case might indicate. Waiting, under these circumstances, is a point which hardly needs to be urged. The leaders of the two movements, we are inclined to think, will recognise the irresistible logic of the actual situation, and fold their hands in, at any rate temporary, peace.

But though the present year must necessarily be one of sitting still, so far as hospital legislation is concerned, it need not be, it must not be, a period of idleness. On the contrary, just as there has never been in the history of hospitals so striking a concurrence of events as that through which we are beginning to pass, and which, we hope, will be brought to so glorious a completion at the close of the year, so must the minds, not of doctors and hospital managers and officials only, but of all the new subscribers, who are thronging into the hospital world, and of all the spectators who may be amazed at the striking and significant spectacle, begin to take earnest thought for all the great and novel circumstances to be anticipated in the near future. The Prince's Fund wlll assuredly effect a peaceful revolution in the hospital kingdom; and the future of medicine and of medical charity may be more noble and serviceable to the State and the world than the most sanguine among us has ever dreamed.

In the meantime effort of every variety, but all as strong and persistent as it can by any possibility be made, should be devoted to the one object of making the fund successful in the highest and most magnificent degree. The days of doubt are over. The Fund is here: it has started on its journey at a gallop, and all men are glowing with enthusiasm. That its object is unsurpassably good, who can question? That it has aroused the sympathy and enthusiasm of all London, and all within reach of London's spreading mantle, is one of the most significant as it is one of the most honourable signs of the times; that the sympathy of the rich with the poor is thereby demonstrated in a way which carries conviction to the most cynical is plain to all men; and that the real and true brotherhood of the classes and the masses could in no other way be so humanely cemented will be obvious to all who think. If we may be permitted to try to put into one sentence the feelings which are, in a daily intensifying degree, quickening and animating all classes, we should say "God Bless the Hospitals' Fund" and "God Bless the Prince of Wales."

